# Digital health competences and AI beliefs as conditions for the practice of evidence-based medicine: a study of prospective physicians in Canada

**DOI:** 10.1080/10872981.2025.2459910

**Published:** 2025-01-31

**Authors:** Gerit Wagner, Mickaël Ringeval, Louis Raymond, Guy Paré

**Affiliations:** a Faculty Information Systems and Applied Computer Sciences, Otto-Friedrich Universität, Bamberg, DE, Germany; b Département de technologies de l’information, HEC Montréal, Montréal, CA, Canada; c Université du Québec à Trois-Rivières, CA

**Keywords:** Digital health, artificial intelligence, evidence-based medicine, medical students, survey

## Abstract

**Background:**

The practice of evidence-based medicine (EBM) has become pivotal in enhancing medical care and patient outcomes. With the diffusion of innovation in healthcare organizations, EBM can be expected to depend on medical professionals’ competences with digital health (dHealth) and artificial intelligence (AI) technologies.

**Objective:**

We aim to investigate the effect of dHealth competences and perceptions of AI on the adoption of EBM among prospective physicians. By focusing on dHealth and AI technologies, the study seeks to inform the redesign of medical curricula to better prepare students for the demands of evidence-based medical practice.

**Methods:**

A cross-sectional survey was administered online to students at the University of Montreal’s medical school, which has approximately 1,400 enrolled students. The survey included questions on students’ dHealth competences, perceptions of AI, and their practice of EBM. Using structural equation modeling (SEM), we analyzed data from 177 respondents to test our research model.

**Results:**

Our analysis indicates that medical students possess foundational knowledge competences of dHealth technologies and perceive AI to play an important role in the future of medicine. Yet, their experiential competences with dHealth technologies are limited. Our findings reveal that experiential dHealth competences are significantly related to the practice of EBM (β = 0.42, *p* < 0.001), as well as students’ perceptions of the role of AI in the future of medicine (β = 0.39, *p* < 0.001), which, in turn, also affect EBM (β = 0.19, *p* < 0.05).

**Conclusions:**

The study underscores the necessity of enhancing students’ competences related to dHealth and considering their perceptions of the role of AI in the medical profession. In particular, the low levels of experiential dHealth competences highlight a promising starting point for training future physicians while simultaneously strengthening their practice of EBM. Accordingly, we suggest revising medical curricula to focus on providing students with practical experiences with dHealth and AI technologies.

## Introduction

Establishing evidence-based medicine (EBM) across medical professions and specialties is essential for reducing costs and improving patient outcomes. Prior research on this topic indicates that EBM results in more efficient resource use, enhanced patient care, decreased costs and hospital stays, increased patient satisfaction, and the elimination of unnecessary or ineffective medical practices [1–3]. The economic benefits of EBM and the corresponding improvements in the quality of care are particularly pronounced in chronic care settings. Advances in digital health (dHealth) and artificial intelligence (AI) technologies promise to further enhance the quality and personalization of healthcare [4]. However, prior research indicates that the successful adoption of evidence-based innovations, such as dHealth and AI, depends on various regulatory, administrative, and human factors [5]. Individual physicians will play a pivotal role in competently applying advanced technologies to support EBM.

Given that dHealth and AI technologies are integral to the future of medicine [6,7], in the present study we focus on the competences of prospective physicians as facilitating conditions for their practice of EBM. The World Health Organization (WHO) has dedicated a global strategy to the promises and challenges associated with dHealth technologies [8], anticipating that these technologies will play a pivotal role. Effectively leveraging these technologies requires ‘more evidence-based knowledge, skills, and competence for professionals to support healthcare’ [8, p.8]. While such competences would significantly improve clinical knowledge management and practices [9], current research has identified a lack of knowledge in this area as a major barrier to effective EBM [10,11].

With regards to medical education, dHealth knowledge and skills are now deemed to matter for medical students, not only as prospective physicians, but as future practitioners of EBM [4]. For instance, this is reflected in their use of evidence-based clinical decision support systems in the course of their medical training [12]. As dHealth technologies are now being used to generate better evidence and deliver evidence-based care [13], these technologies, and AI-related technologies in particular, also play a pivotal role in developing the EBM competences of prospective physicians [14], and in regard to their evidence seeking and evaluation skills in particular [15; 16]).

Prior work has repeatedly emphasized the integral role of dHealth and AI technologies in healthcare, but few studies go beyond general knowledge barriers to EBM. For example, Pravikoff, Tanner, and Pierce [17] found that only 46% of nurses surveyed had prior knowledge of evidence-based nursing practices, while 67% primarily relied on other nurses for information. Similarly, Kaseka and Mbakaya [18] confirmed that general measures of practice, attitude, and knowledge levels predict evidence-based nursing behaviors in midwives. Studies reviewed by Portela Dos Santos et al. [19] also analyzed broad conceptions of knowledge and competence gaps as antecedents of EBM, rather than providing specific insights into the role of technology-related competences. Therefore, there are limited empirical insights into the specific effects of prospective physicians’ dHealth competences as well as their perceptions related to AI technologies. The present study attempts to fill this gap.

More precisely, we aim to answer the following research questions: What is the effect of dHealth competences and attitudes towards AI upon the practice of EBM by prospective physicians? And what is the effect of their individual background upon their level of dHealth competences? Our ensuing research objective is thus to generate new knowledge of the causal relationships between the dHealth competences, the attitudes towards AI, and the EBM practice of medical students within their curriculum. To achieve our objective, we build on prior implementation research frameworks to develop a theoretical model with corresponding hypotheses. The methods section explains the data collection context, situated in a Canadian medical school. The survey administration, measurement instruments, and analytical approach are presented before the principal findings. We then discuss the implications of our study for the medical curriculum, as well as its limitations and promising areas for future research.

## Theoretical model

Our theoretical model builds on prior work to understand medical students’ practice of EBM as advanced dHealth and AI technologies become increasingly significant in their future profession [20]. Evidence-based medical practice involves integrating evidence, clinical judgment, and patient values and preferences to create and apply a customized care plan [18,21]. Understanding this behavioral outcome is essential, given the benefits associated with it, such as improved quality of care, reduced costs, and lower variability of care [22,23].

For prospective physicians, the diffusion of disruptive technologies in the profession, such as advanced AI tools, simultaneously creates concerns (e.g., fear of job loss) and hopes (e.g., new career opportunities, the replacement of laborious tasks, and improved patient service) [24,25]. Additionally, healthcare personnel may perceive AI as augmenting or replacing work in various medical areas, either through new models like deep or personalized medicine [4,26] or through enhanced capabilities for research synthesis [27]. Within the context of digital transformation and the disruptive effects of AI [28], technologies ranging from wearable devices to new AI tools are creating opportunities related to precision medicine and patient empowerment [29,30]. ,These developments raise fundamental questions about the antecedents of prospective physicians’ continued practice of EBM. To our knowledge, existing research has yet to examine how emerging AI technologies and dHealth competences affect their practice of EBM.

Prior mid-level theory explaining EBM-related outcomes can be found in the Consolidated Framework for Implementation Research (CFIR) and the Capabilities, Opportunities, Motivation, and Behavior (COM-B) model. The CFIR is an established framework that includes a broad array of constructs to explain the implementation of evidence-based innovations, such as AI [5]. Among the determinants related to the inner and outer organizational settings and the implementation process, it also features individual characteristics as determinants contributing to the successful adoption of evidence-based innovations in practice [5].

**Figure 1.**
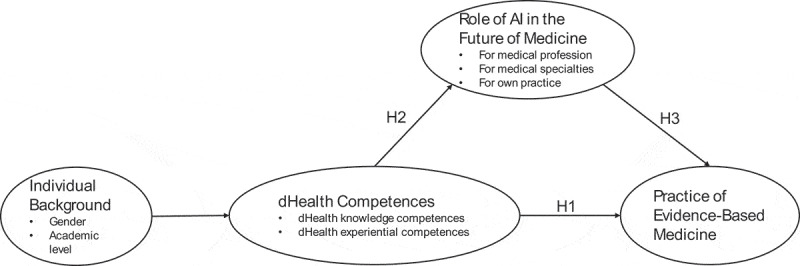
Theoretical model.

Regarding the role of individuals, such as physicians and nurses, CFIR builds on the COM-B model [31], which covers the constructs of needs, capabilities, opportunities, and motivation. According to COM-B, these constructs are key determinants of individuals implementing evidence-based practices as a behavioral outcome. The model has been applied in previous research [32] and validated in comparison to the Theory of Planned Behavior (TPB) by Howlett, Schulz, Trivedi, Troop, and Chater [33]. For our work, COM-B underscores the role of individual capabilities related to technology, suggesting that evidence-based practice can be further influenced by individuals’ motivations and perceptions of opportunities. Below, we develop the hypotheses included in the model, as displayed in Figure 1.

Prior research suggests that knowledge and experiential competences, including those related to dHealth and AI, are directly linked to the practice of EBM. Recent literature reviews confirm that the lack of knowledge and skills is a major barrier to implementing evidence-based practice at the individual level (e.g [19,34]. These barriers persist across healthcare settings, including primary healthcare involving nurses and physiotherapists [19], as well as in developing countries [34]. While published studies have considered general predictors related to EBM knowledge, skills, and competences [19], physicians’ competences to operate dHealth technologies, such as clinical decision support systems, are recognized as critical for integrating patient preferences, which is one of the cornerstones of EBM [35].

Knowledge and experiential competences with regards to dHealth may be conceptualized in terms of the physicians’ familiarity and experimentation with various health technologies and applications such as health IT-based systems (e.g., EHRs), AI-based technologies (e.g., machine learning) and connected medical objects, as well as telehealth (e.g., teleconsultation) and mobile applications [20]. In this regard, we posit that dHealth competences are an essential facet of the knowledge and competences predicting EBM. For example, the study by Kaseka and Mbakaya [18] measures knowledge levels generally, including items such as IT skills and awareness of major information sources. As dHealth technologies continue to transform the way healthcare services are provided to patients, their knowledgeable and competent use is expected to impact EBM practices at the individual level. We therefore formulate the following hypothesis:

Hypothesis 1 (H1): Knowledge and experiential dHealth competences are positively associated with medical students’ practice of EBM.

Complementing prior research, our theoretical model goes beyond competences to consider how prospective physicians perceive the role of AI in the future of medicine as a determinant of evidence-based practice behavior. We take dHealth knowledge and experiential competences as a starting point, referring to the ‘ability to use information retrieved from an electronic source to solve a health problem’ [36, p.2]. While previous work has linked dHealth competences with EBM [18,19], students who acquire substantial dHealth competences in the medical curriculum are expected to be more sensitive to the role that advanced technologies, such as AI, may play in their future profession. To understand this effect, we specify the construct of students’ perceptions of AI in the future of medicine, which refers to their positive or negative perceptions of how AI and associated technologies like machine learning (ML) and natural language processing (NLP) will affect the practice of medicine. Consequently, we state:

Hypothesis 2 (H2): The more knowledge and experiential dHealth competences medicine students have, the more they acknowledge the importance of AI in their future profession.

Finally, we consider the relationship between AI and EBM, which is particularly interesting because the effect of AI on EBM is unclear *ex ante*. Prior work suggests that AI technologies could benefit EBM through data acquisition and analysis [37], enhance clinical trials [4], and contribute to the enhanced synthesis of existing evidence [27,38]. At the same time, there is limited evidence showing whether prospective physicians perceive AI as reinforcing EBM or whether their perceptions of AI lead them away from traditional conceptions of EBM. Consistent with the COM-B model, we thus expect a positive effect and propose:

Hypothesis 3 (H3): The more importance medical students place on the role of AI in their future profession, the more they will be inclined to practice EBM.

In addressing our ancillary research question, the theoretical model also implicitly hypothesizes that medical students’ individual background (e.g., gender and academic level) impact their dHealth knowledge and experiential competences.

## Methodology

### Data collection context and participants

The present study is part of an ongoing research program examining the perceptions, knowledge, and competences of medical students regarding AI technologies and their influence on evidence-based practice. Situated within the University of Montreal’s medical school in Canada, this study employs structural equation modeling (SEM) to analyze the relationships between medical students’ competences with dHealth technologies, their perceptions of the role of AI in their future profession, and their practice of EBM.

The study population includes undergraduate medical students enrolled at the Université de Montréal, totaling approximately 1,400 students. Building on our research program’s prior phases, we surveyed a representative sample of the current enrollment. Data collection consisted of an electronic questionnaire distributed via the medical school’s mailing list. The survey was administered in French and then translated. Participation was voluntary, and students were assured anonymity to encourage candid responses. The survey directed participants to a secure website hosted on the Qualtrics platform, known for its compliance with data privacy laws and robust data security measures.

### Measurement instruments

Given that prior research does not provide established instruments for all constructs in our model, the measurement items were adapted for the most part from the existing literature on evidence-based medicine [39] and digital health [20]. Most survey items are measured either on 5-point Likert scales or dichotomous (yes/no) scales. The medical students’ dHealth knowledge competences were measured as a formative construct by assessing their familiarity with basic IT systems (5 items), telehealth (2 items), AI-related technologies (4 items), and connected medical objects (11 items). Similarly, the dHealth experiential competences construct was measured by assessing students’ experimentation with basic IT systems (5 items), telehealth (2 items), AI-related technologies (4 items), and mobile medical apps (17 items).

Medical students’ perceptions of the role of AI in the future of medicine were measured using three reflective indicators focusing on the medical profession in general (5 items), the different medical specialties (9 items), and the students’ own practice of medicine (8 items). Finally, the students’ practice of EBM was measured through four reflective scales, based on the existing EBM literature relevant to prospective physicians. The measurement instruments are presented in Appendix A.

### Statistical analysis

Our analytical approach employs structural equation modeling (SEM) to test the theoretical hypotheses related to the relationships between medical students’ dHealth competences, perceptions of AI technologies, and their practice of EBM. The structural model was tested to examine the direct and indirect effects of dHealth competences and AI perceptions on EBM practice. SEM’s capability to model complex relationships and handle latent constructs makes it particularly suitable for our main objectives [40]. We used the SEMinR package (v.2.3.2) for the SEM analysis and refined our model to ensure robust, interpretable results [41].

### Ethics approvals

This study was reviewed and approved by the University of Montreal’s ethics committee (#CERSES-19-108-D). All participants provided their consent electronically before commencing the survey, which included a detailed explanation of the study’s purpose, procedures, risks, and benefits. Participant privacy and data integrity were safeguarded throughout the study, in accordance with research ethics standards and relevant data protection laws.

## Results

### Descriptive statistics

Of the 177 participants, 124 (70%) were women and 51 (29%) were men. The remaining students did not respond. Most participants (n = 113, 64%) were in the early stages of their studies (preparatory year and 1st preclinical year), with an average age of 22.9 years (see Table 1). Generally, the students had below-average dHealth knowledge competences ([1.9, 2.5], SD = 0.8), being less familiar with advanced technologies, such as AI or wearables, than with traditional technologies. Their experiential competences were also low, with slightly more opportunities to experiment with traditional technologies, such as basic IT systems (1.8, SD = 0.9), compared to advanced ones like mobile technologies (1.4, SD = 0.5). Despite having low knowledge (2.1, SD = 0.5) and hands-on experience with AI (1.5, SD = 0.6), our respondents considered this technology to play an important role in the future of medicine, particularly for their medical practice (4.8, SD = 2.9).

**Table 1.** Profile of the respondents.

Individual Background		Final sample (*n* = 177)	
		N	%
Academic level	Preparatory year	38	22%
	1st year preclinical	75	42%
	2nd year preclinical	32	18%
	1st year clerkship	18	10%
	2nd year clerkship	14	8%
Gender	Female	124	70%
	Male	51	29%
	Prefer not to reply	2	1%
Age	Mean	22.9	
	Standard deviation (SD)	3.3	
	Minimum	18	
	Maximum	38	

Reliabilities and descriptive statistics for the research constructs are provided in Table 2. The variance inflation factors (VIF) of all our variables are less than 2, showing that multicollinearity is not an issue in our study [42,43]. According to Kock [44], the model can be considered free of common method bias if all VIFs are equal to or lower than 3.3. Common method bias is therefore not an issue in the present study.

**Table 2.** Reliability and descriptive statistics of the research variables.

Research Construct	α	VIF	Mean	SD	Min	Max
*Individual Background* Academic level Gender	–	1.0 1.0	2.4 0.7	1.2-	1 0	5 1
*dHealth Knowledge Competences* Familiarity with basic IT systems Familiarity with telehealth Familiarity with AI-related technologies Familiarity with connected medical objects	0.83 0.58 0.78 0.93	1.6 1.7 1.4 1.2	2.5 2.2 2.1 1.9	0.9 0.8 0.8 0.8	1.0 1.0 1.0 1.0	5.0 5.0 4.8 5.0
*dHealth Experiential Competences* Experimentation with basic IT systems Experimentation with telehealth Experimentation with AI-related technologies Experimentation with mobile applications	0.88 0.73 0.82 0.91	2.7 2.9 1.5 1.2	1.8 1.6 1.5 1.4	0.9 0.7 0.6 0.5	1.0 1.0 1.0 1.0	5.0 5.0 3.8 4.8
*Role of AI in the Future of Medicine* For the medical profession For the medical specialties For own medical practice	0.75 0.83 0.89	1.5 1.4 1.3	3.7 3.5 4.8	0.5 0.6 2.9	1.6 2.0 0.0	5.0 5.0 8.0
Practice of Evidence-Based Medicine To improve learning by consulting the literature To search the Web for relevant sources To be on the lookout for practice guidelines To take a critical look at the medical literature	–	1.9 1.6 1.9 1.9	3.2 3.9 3.2 3.6	1.1 0.9 1.0 1.0	1.0 1.0 1.0 1.0	5.0 5.0 5.0 5.0

## Measurement model

In the component-based approach to SEM taken in this study, i.e., partial least-squares (PLS), the first step in the analysis is to simultaneously evaluate the measurement model and the research model. Here, one may note that two research constructs, namely, dHealth Knowledge Competences and dHealth Experiential Competences, are modeled as being formative due to the composite and multidimensional nature of their conceptualization [45], whereas the other two constructs, Role of AI in the Future of Medicine and Practice of Evidence-Based Medicine, are reflective [46]. The measurement model also includes another formative construct, Individual Background, made up of two control variables, namely, gender and academic level.

As presented in Table 3, the composite reliability coefficient of the two reflective constructs was equal to 0.85 and 0.97 respectively, above the 0.70 threshold and thus confirming these constructs’ reliability. Also confirmed is these constructs’ convergent validity as their average variance extracted (AVE) was equal to 0.64 and 0.89 respectively, above the 0.50 threshold. The last property to be analyzed in the measurement model, discriminant validity, indicates the extent to which a construct differs from other constructs in the model. In the case of reflective constructs, the shared variance between such a construct and other constructs must be less than its AVE, as confirmed in Table 3. In the case of the three formative constructs, the fact that each shares less than 70% variance with the other constructs in the measurement model, and thus correlates less than perfectly with these constructs, is an indication of such validity [47].

**Table 3.** Reliability, unidimensionality and discriminant validity of the research constructs.

Research Construct	c.r^.a^	AVE^b^	Correlations^e^ 1. 2. 3. 4. 5.				
1. Individual Background	–	–	–^d^				
2. dHealth Knowledge Competences	–	–	0.24	–			
3. dHealth Experiential Competences	–	–	0.45	0.68	–		
4. Role to AI in the Future of Medicine	0.85	0.64	−0.01	0.33	0.39	0.80	
5. Practice of EBM	0.97	0.89	0.03	0.45	0.44	0.43	0.94

^a^composite reliability= (Σλ_i_)^2^/((Σλ_i_)^2^+Σ(1-λ_i_^2^))[inappropriate for formative constructs].

^b^average variance extracted = Σλ_i_^2^/n[“ “ “ “].

^c^loading of the item on its associated construct.

^d^diagonal: (AVE)^1/2^ = (Σλ_i_^2^/n)^1/2^[“ “ “ “].

^e^sub-diagonals: correlation = (shared variance)^1/2^.

## Research model

**Figure 2.**
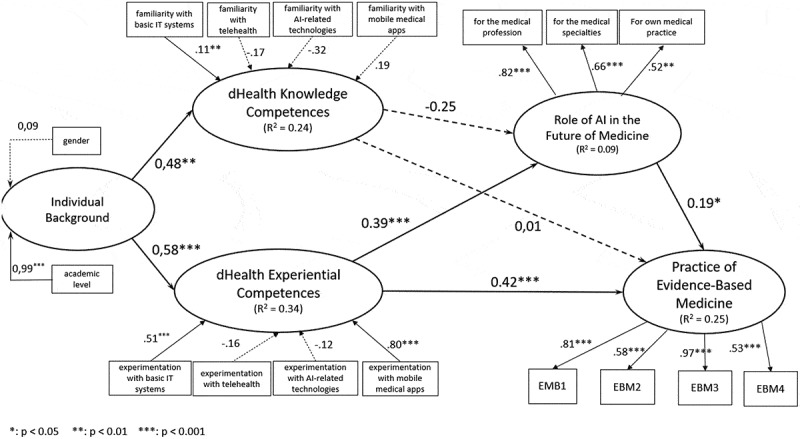
Research model.

The results of testing the research model through PLS-SEM analysis are presented in Figure 2. This first shows that medical students’ individual background, characterized by gender and academic level, has a significantly positive effect on both components of their dHealth competences (knowledge competences: β = 0.48, *p* < 0.01; experiential competences: β = 0.58, *p* < 0.001). This finding suggests that personal academic achievement and possibly gender-related factors contribute to an individual’s ability to understand and apply dHealth knowledge in practice.

The construct of experiential dHealth competences was also strongly correlated with the practice of EBM (β = 0.42, *p* < 0.001), while dHealth knowledge competences were not significantly related to the dependent variable (β = 0.01, *p* > 0.05, not significant), offering partial support for Hypothesis 1 (H1). Additionally, dHealth experiential competences were correlated with the perceived role of AI in the future of medicine (β = 0.39, *p* < 0.001), suggesting that a deeper understanding of dHealth may foster more optimistic views on the potential of AI in medicine. Interestingly, dHealth knowledge competences did not have a significant effect on students’ AI perceptions, providing partial support for Hypothesis 2 (H2). Lastly, the practice of EBM was positively influenced by the perceived role of AI in the future of medicine (β = 0.19, *p* < 0.05), supporting Hypothesis 3 (H3). However, the influence of the perceived role of AI on the practice of EBM is less pronounced compared to the influence of dHealth experiential competences (β = 0.42, *p* < 0.001). This implies that while the anticipation of AI’s role plays a substantial part in the practical implementation of EBM, experiential competences in dHealth have a stronger impact on medical students’ EBM behavior. Overall, our research model explains 25% of the variance in the practice of EBM. The explanatory power is particularly notable, suggesting that both competences in dHealth and attitudes towards AI technology play a critical role in shaping evidence-based practice in medicine.

In summary, our findings suggest that there is a multifaceted relationship between dHealth competences, and perceptions towards AI that collectively influence the practice of EBM. They underscore the importance of integrating dHealth competences into medical education and practice to better prepare prospective physicians for an increasingly technology-driven healthcare environment.

## Discussion

### Principal findings

The principal findings of our study advance the current understanding of how medical students’ competences and perceptions of the role of AI in their future profession relate to their practice of EBM. Given the limited empirical research on these relationships, we conducted an exploratory study to provide initial insights into whether students’ competences and perceptions of advanced technologies lead them to abandon or reinforce the tradition of using recent research evidence to guide their medical practices. Specifically, we find that competences and perceptions of dHealth and AI technologies are positively related to medical students’ EBM behavior. This insight is valuable for medical practice, as it suggests potential synergies between advanced technologies and EBM and highlights that different competences can be taught within the medical curriculum.

Regarding the role of different forms of dHealth competences, it is particularly instructive to appreciate how the effects of knowledge and experiential competences differ. The analyses confirm a significant positive effect of experiential competences, while knowledge competences were not found to be related to EBM. Thus, there is an essential difference between familiarizing medical students with concepts and giving them the opportunity to experiment with IT systems, telehealth applications, AI-related technologies, and mobile medical apps. Additionally, our findings indicate that individual backgrounds, particularly gender and academic level, significantly impact dHealth competences, including both knowledge and experiential aspects. This highlights the necessity of integrating dHealth competences into medical education and practice to accommodate varying individual backgrounds and technological advancements.

### Implications

Healthcare providers face the challenge of leveraging advanced dHealth and AI technologies to simultaneously reduce costs and enhance the quality of care. The successful implementation of technological innovations in practice depends on individual physicians, who interact with patients and deliver healthcare services in line with state-of-the-art evidence and best practices.

First, our work shows that students’ dHealth competences and perceptions of AI – which were previously identified as areas where students had limited knowledge [48–50] – could also make a positive contribution to EBM. This insight is particularly valuable given the benefits of EBM for quality of care [51] and considering that the implementation of technological innovations depends on the capabilities, motivations, and opportunities of individuals [31]. It implies that strengthening education in the areas of dHealth and AI in medical curricula may simultaneously strengthen students’ EBM and offer an effective basis for the technology-supported future of medicine.

Second, we show that the type of dHealth competences matters, in that experiential competences have significant positive effects, while pure knowledge competences were not significant. A direct implication for medical educators is the need to go beyond pure traditional teaching formats to convey knowledge, and to develop teaching approaches that offer realistic, experiential learning opportunities. Specifically, traditional teaching of conceptual and theoretical knowledge could be complemented by novel and interdisciplinary formats to convey experiential knowledge, such as ‘hackathons’ or capstone projects [20]. Such individual or group-based projects are a common element in technical or applied disciplines, such as computer science and information systems. This approach will prepare prospective physicians to effectively integrate advanced technologies into their practice, ultimately supporting the widespread adoption of EBM. While prior research on medical education and AI has highlighted the need to go beyond traditional approaches of delivering teaching contents [52], it is evident that faculty members at medical schools need to acquire requisite dHealth competences, e.g., through interdisciplinary initiatives, training, or hiring [53].

Third, some experiential competences are more important than others, indicating how teaching could prioritize areas. It is noteworthy that more weight is observed on the items related to experimentation with mobile apps and basic IT systems, compared to telehealth technologies or AI-based systems. As such, it is advisable to cover fundamental dHealth technologies broadly instead of focusing on AI exclusively. This aligns with prior curriculum development efforts, which situate AI contents as an advanced topic after covering basic data literacy or IT infrastructure topics [54]. Medical educators who include AI in their courses may expect a good resonance, given that students generally support more formal AI training [48,55,56].

Fourth, our work has implications for the integrated development of AI-related and EBM-related competences within the medical curriculum. Now, these two types of competences are gradually developing a symbiotic relationship, as physicians’ clinical reasoning and practice are increasingly bound by AI-based evidence (and by the limitations associated with such evidence) [57]. Indeed, propositions for medical curricula that emphasize the links between AI and EBM have emerged [58]. Our findings thus constitute both a theoretical and an empirical foundation for such propositions, as evidence generation and evidence synthesis are increasingly enabled by AI and machine learning [59], and as AI becomes a mean for prospective physicians to overcome the barriers encountered in their learning and practice of EBM [60].

### Limitations and future research

This study presents a number of limitations that highlight avenues for future research.

First, our investigation was confined to a single Canadian medical school, limiting the transferability of our results to different contexts in medical education. This is particularly relevant when considering variations in physician career trajectories, levels of country development, healthcare systems, and medical profession regulatory environments. Second, despite our intention for parsimony, the theoretical framework could be broadened in subsequent studies to incorporate additional factors such as social influence and effort expectancy, aligning more closely with earlier IT-related behavioral research. Furthermore, the scope could also be expanded to encompass not only dHealth technologies and applications but also IT-enabled capabilities in medical knowledge management. This would include areas such as e-healthcare intelligence and e-collaboration, which are essential for prospective physicians to engage effectively in contemporary medical practices and to remain both innovative and productive.

Additionally, the measurement items were for the most part specifically designed for this study, using general terminology like AI, ML, and big data analytics. Future studies could use our instrument as a foundation to craft more comprehensive operational definitions, extending beyond AI in healthcare, and validate them empirically. Lastly, as causality cannot be inferred from a cross-sectional (observational) research design, future studies rather using a longitudinal (interventional) design could explore how AI-related educational interventions might influence the choice of research variables. Medical education researchers could also explore how such interventions might influence students’ choices of medical specialties by focussing on specific impacts of AI on medical practice.

## Conclusion

In their future practice, medical students will be expected to concomitantly practice EBM and work with dHealth and AI technologies, which have the potential to improve quality, access, and cost of care. Our study shows that students’ dHealth competences and perceptions of the role of AI in the future of medicine are positively associated with their practice of EBM. Consequently, incorporating advanced technologies into the medical curriculum can simultaneously enhance students’ knowledge and experiential competences, shape their perceptions of AI, and support their practice of EBM. We hope that our findings will help medical schools design a curriculum that better integrates the dHealth, AI and EBM competences of prospective physicians and thus leads to improve healthcare outcomes for their future patients.

## Appendix A.

### Appendix A. Measurement of the research variables

#### dHealth Knowledge Competences

*What is your level of familiarity with the following technologies and applications ?*

**Table:**

	Very low (1)	Low (2)	Moderate (3)	High (4)	Very high (5)
**Familiarity with basic IT systems** with electronic medical records (EMR) with electronic health records (EHR) with *Dossier santé Québec* with *Carnet santé Québec* with *Rendez-vous santé Québec*	□ □ □ □ □	□ □ □ □ □	□ □ □ □ □	□ □ □ □ □	□ □ □ □ □
**Familiarity with telehealth** with teleconsultation with tele-expertise	□ □	□ □	□ □	□ □	□ □
**Familiarity with AI-related technologies** with artificial intelligence with machine learning with big data with Internet of things	□ □ □ □	□ □ □ □	□ □ □ □	□ □ □ □	□ □ □ □

#### Familiarity with connected medical objects

*What is your level of familiarity with the following connected medical objects ?*

**Table:**

	Very low (1)	Low (2)	Moderate (3)	High (4)	Very high (5)
Familiarity with: connected tensiometer connected pulse oximeter connected thermometer connected glucometer connected ophthalmoscope connected autorefractometer connected miniature electrocardiogram connected otoscope connected spirometer connected stethoscope connected ultrasound probe	□ □ □ □ □ □ □ □ □ □ □	□ □ □ □ □ □ □ □ □ □ □	□ □ □ □ □ □ □ □ □ □ □	□ □ □ □ □ □ □ □ □ □ □	□ □ □ □ □ □ □ □ □ □ □

#### dHealth Experiential Competences

*To what extent have you been exposed to the following technologies and applications in the course of your medical education and training ?*

**Table:**

	Not at all (1)	A little (2)	Somewhat (3)	Enough (4)	Very much (5)
**Experimentation with basic IT systems** with electronic medical records (EMR) with electronic health records (EHR) with *Dossier santé Québec* with *Carnet santé Québec* with *Rendez-vous santé Québec*	□ □ □ □ □	□ □ □ □ □	□ □ □ □ □	□ □ □ □ □	□ □ □ □ □
**Experimentation with telehealth** with teleconsultation with tele-expertise	□ □	□ □	□ □	□ □	□ □
**Experiment. with AI-related technologies** with artificial intelligence with machine learning with big data with Internet of things	□ □ □ □	□ □ □ □	□ □ □ □	□ □ □ □	□ □ □ □

#### Experimentation with mobile applications

*How frequently have you been exposed to the following mobile applications in the course of your medical education and training ?*

**Table:**

	Never (1)	Rarely (2)	Regularly (3)	Often (4)	Very often (5)
Experimentation with: UpToDate BMJBestPractice Calculate by QxMD ClinicalKey DxSaurus DynaMed Mobile Epocrates INESSS IPharmacy Lanthier MDCalc MedCalx MedPage Today Medscape NEJM This Week Omnio Pepid other:	□ □ □ □ □ □ □ □ □ □ □ □ □ □ □ □ □ □	□ □ □ □ □ □ □ □ □ □ □ □ □ □ □ □ □ □	□ □ □ □ □ □ □ □ □ □ □ □ □ □ □ □ □ □	□ □ □ □ □ □ □ □ □ □ □ □ □ □ □ □ □ □	□ □ □ □ □ □ □ □ □ □ □ □ □ □ □ □ □ □

#### Role of AI in the future of medicine

**For the medical profession**

*In your opinion, how will artificial intelligence and its components (machine learning, deep learning, etc.) affect each of the following aspects of the future of medicine ?*

**Table:**

	Very negative (1)	Rather negative (2)	No effect (3)	Rather positive (4)	Very positive (5)
Effect on: Prevention of diseases Diagnosis of diseases Treatment of diseases Prognosis of diseases Patient-physician relation	□ □ □ □ □	□ □ □ □ □	□ □ □ □ □	□ □ □ □ □	□ □ □ □ □

#### For the medical specialties

*In your opinion, to what extent will the following medical specialties be affected by artificial intelligence in the future ?*

**Table:**

	Not at all (1)	A little (2)	Somewhat (3)	Enough (4)	Very much (5)
Effect on: Pathology Radiology Dermatology Ophthalmology Emergency and critical care Family medicine Internal medicine Psychiatry Surgery	□ □ □ □ □ □ □ □ □	□ □ □ □ □ □ □ □ □	□ □ □ □ □ □ □ □ □	□ □ □ □ □ □ □ □ □	□ □ □ □ □ □ □ □ □

#### For own medical practice

*Do you plan to use artificial intelligence after your studies to perform the following tasks in your medical practice ?*

**Table:**

	Yes (1)	No (0)
Use of artificial intelligence to: Analyze images of a radiologic nature Analyze images of a photographic nature (e.g., eye fundus) Analyze images of a pathologic nature (e.g., biopsy specimen) Make diagnoses regarding patients Make prognoses regarding patients Determine patient care protocols Analyze data from my anamnesis to generate an opinion Supervise and evaluate patient interviews	□ □ □ □ □ □ □ □	□ □ □ □ □ □ □ □

#### Practice of evidence-based medicine

*To what extent have you adopted the following practices in the course of your medical education and training ?*

**Table:**

	Never (1)	Rarely (2)	Sometimes (3)	Regularly (4)	Very often (5)
I consult scientific literature in order to improve my learning of medicine, beyond the obligatory texts. I conduct research on the web to identify relevant and credible sources that can contribute to my medical training. I am on the lookout for guidelines for the practice of medicine. I take a critical look at scientific and professional literature related to medicine.	□ □ □ □	□ □ □ □	□ □ □ □	□ □ □ □	□ □ □ □
